# Linking Arrhythmias and Adipocytes: Insights, Mechanisms, and Future Directions

**DOI:** 10.3389/fphys.2018.01752

**Published:** 2018-12-05

**Authors:** Maria A. Pabon, Kevin Manocha, Jim W. Cheung, James C. Lo

**Affiliations:** ^1^Joan and Sanford I. Weill, Department of Medicine, New York Presbyterian Hospital, Weill Cornell Medicine, New York, NY, United States; ^2^Division of Cardiology, Department of Medicine, New York Presbyterian Hospital, Weill Cornell Medicine, New York, NY, United States; ^3^Metabolic Health Center, Weill Cornell Medicine, New York, NY, United States; ^4^Department of Pharmacology, Weill Cornell Medicine, New York, NY, United States

**Keywords:** arrhythmia, obesity, adipocyte, fat, atrial fibrillation, epicardial adipose tissue, intramyocardial adipose

## Abstract

Obesity and atrial fibrillation have risen to epidemic levels worldwide and may continue to grow over the next decades. Emerging evidence suggests that obesity promotes atrial and ventricular arrhythmias. This has led to trials employing various strategies with the ultimate goal of decreasing the atrial arrhythmic burden in obese patients. The effectiveness of these interventions remains to be determined. Obesity is defined by the expansion of adipose mass, making adipocytes a prime candidate to mediate the pro-arrhythmogenic effects of obesity. The molecular mechanisms linking obesity and adipocytes to increased arrhythmogenicity in both the atria and ventricles remain poorly understood. In this focused review, we highlight areas of potential molecular interplay between adipocytes and cardiomyocytes. The effects of adipocytes may be direct, local or remote. Direct effect refers to adipocyte or fatty infiltration of the atrial and ventricular myocardium itself, possibly causing increased dispersion of normal myocardial electrical signals and fibrotic substrate of adipocytes that promote reentry or adipocytes serving as a direct source of aberrant signals. Local effects may originate from nearby adipose depots, specifically epicardial adipose tissue (EAT) and pericardial adipose tissue, which may play a role in the secretion of adipokines and chemokines that can incite inflammation given the direct contact and disrupt the conduction system. Adipocytes can also have a remote effect on the myocardium arising from their systemic secretion of adipokines, cytokines and metabolites. These factors may lead to mitochondrial dysfunction, oxidative stress, autophagy, mitophagy, autonomic dysfunction, and cardiomyocyte death to ultimately produce a pro-arrhythmogenic state. By better understanding the molecular mechanisms connecting dysfunctional adipocytes and arrhythmias, novel therapies may be developed to sever the link between obesity and arrhythmias.

## Introduction

**Figure 1:**
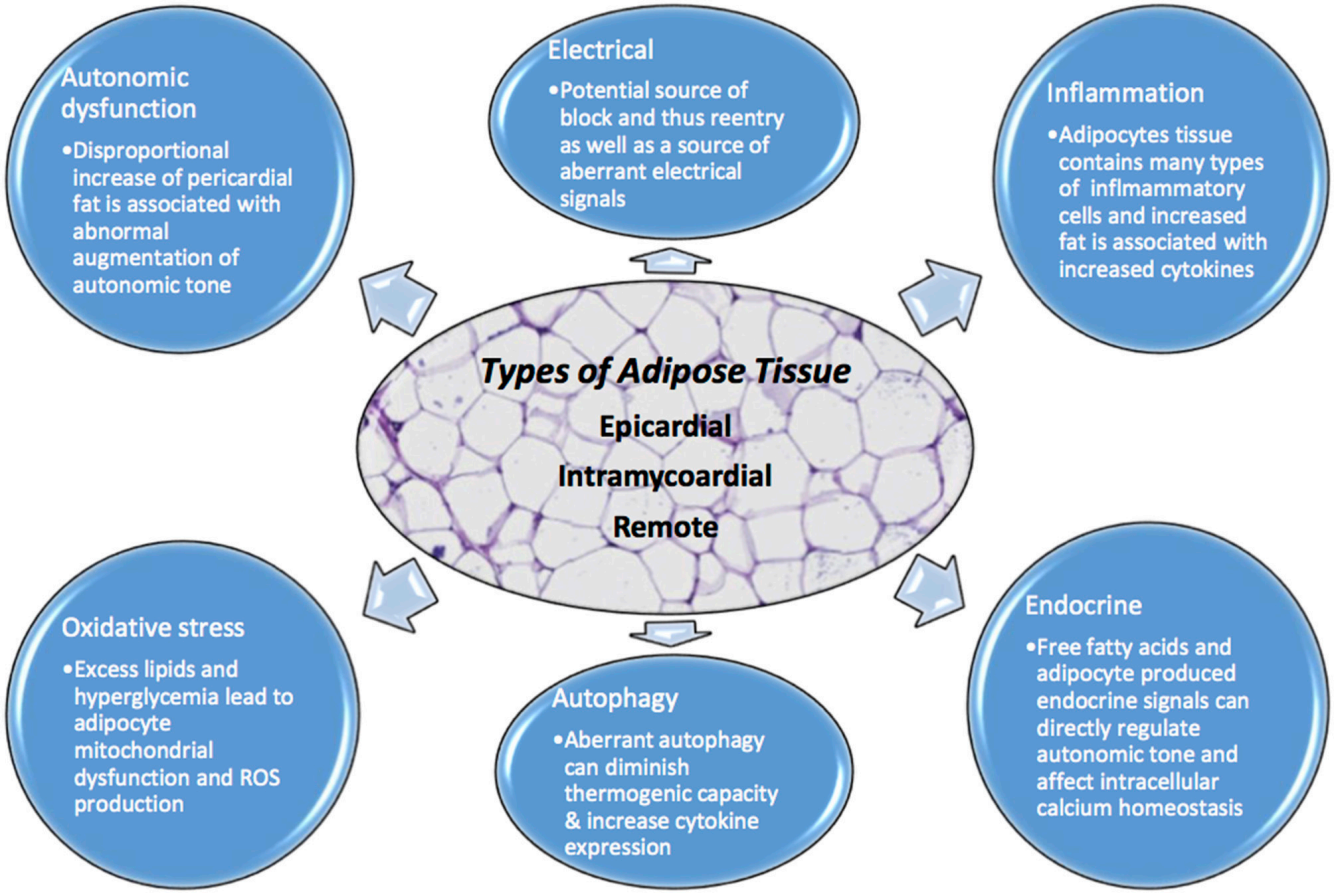
Adipocytes have numerous physiologic effects on myocytes including but not limited to endocrine, inflammatory, autonomic regulation, oxidative, autophagy, and electrical. When homeostasis is dysregulated due to excess adipocyte burden, no matter where the location, there is opportunity for arrhythmia. Excessive adipose tissue can be a source of increased ROS, aberrant autophagy, intracellular calcium dysregulation, autonomic tone augmentation, a cause of block and reentry among others.

From 1980 to 2015, obesity rates at the population level have been steadily on the rise (Afshin et al., 2017). The association between obesity and hypertension, obstructive sleep apnea and diabetes is already well-known. Historically, fat has been an underappreciated organ. Much progress has been made in the last decades to elucidate the multiple functions of fat (Rosen and Spiegelman, 2014). These include endocrine functions such as control of nutritional intake (Halberg et al., 2008; Coelho et al., 2013), inflammatory functions including the production of cytokines and chemokines as well as electrical functions such as the presence of gap junctions (Linck et al., 1974). This growing knowledge along with the rise of obesity to epidemic proportions (Afshin et al., 2017) has led investigators to explore the relationship between obesity and cardiovascular disease. Specifically, of interest to us, is the relationship between obesity and arrhythmias. The links between obesity and atrial fibrillation specifically have been reviewed elsewhere (Lavie et al., 2017); however the molecular mechanisms of how fat may drive arrhythmias, both in the atria and ventricles have not been well-described. This state-of-the-art review will summarize the pathophysiologic molecular mechanisms by which obesity can lead to arrhythmia (Figure 1).

## Types of Fat and Arrhythmia Development

### Local Adipose Tissue

The most studied fat depot linked to arrhythmia is epicardial adipose tissue (EAT). Firstly, the presence of some adipose tissue in and around the heart is physiologic. Fat around the heart can be divided into pericardial adipose tissue and visceral EAT. Pericardial fat is defined by fat surrounding the parietal pericardium while visceral EAT is defined by fat between the myocardium and visceral pericardium (Tam et al., 2016). EAT covers 80% of the heart and is present mostly along the coronary arteries, in the atrioventricular/intraventricular grooves, over the right ventricle (three or four times more than the left ventricle) especially along the right border, anterior surface and at the apex. Physiologic functions include buffering the coronary arteries against torsion and facilitating coronary artery remodeling. EAT has higher fatty acid synthesis and breakdown compared to many other fat depots (Marchington and Pond, 1990), which may serve to protect the heart against high levels of circulating lipids and as reserve in case of increased physiologic demand (i.e., exercise or infection; Iacobellis and Barbaro, 2008).

Although EAT is a type of visceral adipose tissue (VAT), it has unique characteristics such as smaller adipocytes (Bambace et al., 2014), different fatty acid composition, higher protein content and lower rates of glucose utilization (Iacobellis et al., 2005a; Iacobellis and Barbaro, 2008). The brown fat protein, uncoupling protein-1 (UCP-1), has been found to be expressed at high levels in EAT, suggesting a possible role in heart thermogenesis (Sacks et al., 2009). However, as with other homeostatic processes in the human body, there is a fine balance between physiologic quantity of EAT vs. excess. For instance, EAT accumulation may be part of a compensatory mechanism developed in response to chronic insults of the myocardial tissue, to support its higher demand-metabolic condition with free fatty acids, but this response can lead to increased inflammation and fibrosis to create a substrate for arrhythmias (Suffee et al., 2017).

Clinical imaging studies have demonstrated a strong direct correlation between epicardial adipose tissue (EAT) and abdominal visceral adiposity. EAT thickness has been reported to be associated with severity of coronary artery disease (CAD) (Jeong et al., 2007), atrial fibrillation (AF) recurrences after catheter ablation (Tsao et al., 2011), and adverse cardiovascular (CV) outcomes associated with AF (Chu et al., 2016). EAT and pericardial fat quantified by magnetic resonance imaging (MRI) is associated with the development of ventricular tachycardia (VT), ventricular fibrillation (VF) and long-term overall mortality in patients with systolic heart failure (HF; Wu et al., 2015). In an Asian population, pericardial fat thickness was also associated with increased frequency of premature ventricular contractions (Tam et al., 2016).

Multiple possible mechanisms could explain these findings. In the presence of obesity or metabolic syndrome, adipose tissue develops different characteristics resulting in changes such as “whitening” of the adipose tissue embedded within or surrounding the myocardial tissue. The adipocytes become larger in size with increased expression of inflammatory markers and decreased adiponectin levels (Bambace et al., 2014; Song et al., 2015; Zghaib et al., 2016). This pro-inflammatory environment likely leads to recruitment of inflammatory cells to perpetuate inflammation, leading to fibrosis (Cherian et al., 2012). Moreover, EAT shares the same coronary blood supply as the adjacent ventricular myocardium and has direct contact with it given the lack of fascia between adipocyte and myocardial layers (Guglielmi and Sbraccia, 2017). Therefore, EAT can have paracrine effects and directly infiltrate myocardium affecting cardiac function. Gap junctions have also been demonstrated within epicardial fat, which may lead to abnormal electrical coupling between the myocardium and EAT (Linck et al., 1974). For example, peri-atrial EAT has been found to express genes implicated in oxidative phosphorylation, muscular contraction, and calcium signaling in AF patients, which could potentially increase the translocation of cytosolic calcium into the lumen of the sarcoplasmic reticulum promoting excitation-contraction coupling and thus lead to disruptive electrophysiologic signals (Gaborit et al., 2015). Specific mechanisms associating EAT with development of arrhythmias are discussed below.

#### Inflammation and EAT

It is well-known that obesity is accompanied by a state of chronic low-grade inflammation. Adipose tissue contains different cell types other than adipocytes such as preadipocytes, macrophages, stromal cells, endothelial cells, and lymphocytes. The immune cells present within the adipose tissue can release a variety of chemokines, cytokines, and other factors contributing to pathogenic inflammation (Wong et al., 2017). In patients with CAD, EAT has been shown to have increased levels of inflammatory cytokines (Mazurek et al., 2003; Baker et al., 2009; Hirata et al., 2011a; Zhou et al., 2011; Greulich et al., 2012; Vianello et al., 2016) along with augmented number of pro-inflammatory M1 macrophages (Hirata et al., 2011a; Vianello et al., 2016) and T cells (Hirata et al., 2011b). Adiponectin expression in EAT (a potent anti-inflammatory adipokine) seems to be lower in patients with CAD compared to non-CAD patients (Iacobellis et al., 2005b; Zhou et al., 2011).

This greater inflammatory environment can provide substrate for arrhythmia development. In fact, increased EAT levels of adiponectin are related to decreased risk of developing post-operative AF in patients undergoing cardiac surgery (Kourliouros et al., 2011). Specifically, peri-atrial EAT seems to play an important role in the development of AF. Patients with permanent AF have increased EAT volume and serum inflammation biomarkers compared to paroxysmal AF (Nagashima et al., 2012). This correlates with the fact that inflammatory activity of left peri-atrial, atrioventricular groove, and left main artery EAT is greater than in subcutaneous or visceral thoracic tissue from patients with AF compared to controls (Mazurek et al., 2014). Consistently, peri-atrial EAT volume estimated by computer tomography is a predictor of new-onset AF in patients with CAD (Nakanishi et al., 2012). Inflammation of EAT around the LA, determined by higher density in computed tomography images, has been related to the presence of paroxysmal AF (Kusayama et al., 2016).

Recently, it has become evident that different adipose tissue depots, have distinct pro-inflammatory properties (Packer, 2018). The secretory profile pattern of EAT seems to be different than that of pericardial or subcutaneous adipose tissues (SAT) even in the same patient (Greulich et al., 2012). Specifically, EAT samples from patients with CAD have evidence of increased pro-inflammatory markers [NF-κB, IKKβ, and JNK, TNF-α, angiotensinogen, monocyte chemotactic protein (MCP-1), IL-1β, IL-6, sIL-6R] compared to SAT from the same patient (Mazurek et al., 2003; Baker et al., 2009; Hirata et al., 2011a; Zhou et al., 2011; Greulich et al., 2012; Vianello et al., 2016). Samples of EAT from patients paired for cardiovascular, CAD and AF risk factors show that the transcriptomic signature of peri-ventricular EAT appears to be more linked to inflammation in comparison to peri-atrial and peri-coronary EAT (Gaborit et al., 2015). Thus, adipose tissue function is widely heterogeneous, differing both in distinct locations in the body and various regions within the heart.

The potential harmful and pro-fibrotic effects of EAT are suggested by secretome analyses, identifying adipocytokines such as activin A, a member of the transforming growth factor- β (TGF-β) superfamily, which may promote atrial fibrosis (Venteclef et al., 2015). Additionally, rat cardiomyocytes treated with conditioned media obtained from human EAT from patients with diabetes vs. patients without diabetes demonstrate reductions in contractile dysfunction, decreased insulin-mediated Akt-Ser473-phosphorylation (activation of cell survival pathway) and elevations in SMAD2 phosphorylation/activation (a TGF-β pathway protein implicated in cardiomyocyte fibrosis) demonstrating how EAT can affect cardiomyocyte function and remodeling (Greulich et al., 2012). The same study also showed reductions in cytosolic Ca^2+^ fluxes and expression of SERCA2a in rat cardiomyocytes treated with conditioned media from diabetic patients, implicating altered calcium homeostasis that could lead to arrhythmias.

#### Oxidative Stress and EAT

Excessive production of mitochondrial reactive oxygen species (ROS) in adipose tissue is known to be an early instigator of adipose tissue inflammation (Yeop Han et al., 2010; Han, 2016). Increased lipid levels and hyperglycemia can lead to adipocyte mitochondrial dysfunction and ROS production, and this phenomenon has been associated with insulin resistance (Furukawa et al., 2004; Houstis et al., 2006; Yeop Han et al., 2010). This is evident by the fact that obese patients with type 2 diabetes mellitus seem to have increased production of ROS and decreased activity of anti-oxidant enzymes in SAT compared to controls (Chattopadhyay et al., 2015).

Few studies have looked into oxidative stress in EAT in the setting of obesity. As mentioned before, EAT seems to have a thermogenic function given its high expression of UCP-1 (Sacks et al., 2009). Cultured adipocytes isolated from EAT show that the thermogenic phenotype is negatively correlated with the oxidative stress-related parameters at the gene expression level (Chechi et al., 2017), corresponding to the whitening of EAT seen in patients with cardiovascular diseases.

In patients with cardiovascular diseases, there is increased ROS production in EAT compared to SAT, as well as lower catalase levels (Salgado-Somoza et al., 2010). Also, the SAT and EAT from the same patient differ in the post-translational modifications of proteins involved in oxidative stress such as protein disulfide isomerase A1 (PDIA1), glutathione S-transferase P1 (GSTP1), and phosphoglycerate mutase (PGAM1) (Salgado-Somoza et al., 2010). There are transgenic and knockout models of arrhythmias, such as transgenic mice with cardiac-specific overexpression of peroxisome proliferator-activated receptor gamma-1 (PPARγ1) which induces spontaneous ventricular arrhythmias (Morrow et al., 2011). However, ROS production can have beneficial properties as well. In CAD patients cardiomyocytes release products of oxidation that likely activate PPARγ-dependent upregulation of adiponectin expression in EAT, which can act as a defense mechanism (Antonopoulos et al., 2016).

Oxidative stress may play a role in the development of arrhythmias. For instance, in AF, oxidative stress has been correlated to early electrophysiological remodeling (Carnes et al., 2001). AF patients have been described to have higher levels of circulating oxidative stress markers (Neuman et al., 2007), and have upregulation of gene expression associated to oxidative stress (Kim et al., 2003). Proteins involved in myocyte contractility such as myofibrillar creatine kinase (MM-CK), have decreased activity in AF patients related to oxidative injury (Mihm et al., 2001). Anti-oxidant agents may have a therapeutic role (Carnes et al., 2001; Ozaydin et al., 2008). No studies have been done correlating oxidative stress in EAT and AF.

It is important to note that even though ROS are usually seen as deleterious. Under normal conditions, mitochondrial ROS production is increased during brown adipose tissue thermogenesis. In this case, ROS may function as signaling molecules that activate UCP-1 and abrogation of ROS production can lead to induction of hypothermia (Chouchani et al., 2016). Given that EAT has been considered brown adipose tissue with thermogenic properties, it is possible that under non-pathologic conditions, ROS are involved in EAT signaling for thermogenesis regulation as well as adiponectin expression. However, in the setting of obesity, uncontrolled ROS production can elicit inflammation.

#### Autophagy and EAT

Autophagy is a homeostatic process that recycles intracellular components through lysosomal degradation (Mizushima et al., 2008). The majority of the studies have shown that in the setting of obesity, autophagy may be upregulated in adipocytes, but downregulated in the liver, heart, and pancreas (Zhang et al., 2018). There is contradicting evidence regarding the beneficial and deleterious effects of autophagy in obesity and cardiovascular diseases. This is due in part to the fact that results from studies of autophagy regulation may vary depending on the model used, the organ and specific cell evaluated and the chronicity of the stimuli (Pabon et al., 2016; Zhang et al., 2018). Most of the evidence suggests there is decreased autophagic activity in obesity-related metabolic cardiomyopathy (Xu et al., 2013; An et al., 2017; Hu and Zhang, 2017). Aberrant autophagy activation in adipocytes leads to defective browning of the adipose tissue diminishing its thermogenic capacity (Okla et al., 2015), as well as increasing pro-inflammatory cytokine expression (Jansen et al., 2012). Under high fat diet (HFD) conditions, murine adipocytes have increased autophagic activity, leading to degradation of the anti-inflammatory adipokine adiponectin (Zhou and Liu, 2010), suggesting a role of adipocyte-autophagy contributing to the low grade inflammatory state in obesity. Few studies have looked into autophagy activity in EAT. In one study comparing EAT and SAT samples from heart failure patients, EAT was found to have higher expression of genes related to autophagy and mitophagy (selective autophagy of the mitochondria), as well as protein expression related to autophagy (Beclin-1 and microtubule-associated proteins 1A/1B light chain 3B (LC3B-II)) (Burgeiro et al., 2018).

There is limited data regarding the relationship between autophagy and arrhythmia pathogenesis. Autophagy has been shown to be increased in cardiomyocytes in a canine model of AF (tachypacing; Yuan et al., 2014; Wiersma et al., 2017). Furthermore, autophagy seems to be activated by increased endoplasmic reticulum (ER) stress in the setting of AF and inhibition of ER stress can prevent autophagic activity conferring protection for atrial remodeling associated with AF (Wiersma et al., 2017).

In humans, patients with persistent AF have increased autophagosome formation in atrial myocardium determined by electron microscopy and higher levels of LC3B-II compared to patients with normal sinus rhythm (Wiersma et al., 2017) which correlates with previous findings of increased LC3BII/I ratio (marker of autophagic activity) in samples from atrium from AF patients (Yuan et al., 2014). Contrary to these results, in a study using post-operative AF patients atrial samples, there was reduced levels of LC3B-II possibly suggesting impaired autophagic activity (Garcia et al., 2012). Also, higher expression of Beclin-1 and LC3BII has been reported in myocardial muscle of mouse hearts with ventricular fibrillation after ischemia reperfusion injury, compared to non-fibrillated hearts (Meyer et al., 2013). The interpretation of these findings is difficult given that these could be secondary to increased autophagic flux leading to consumption of LC3B-II or decreased autophagy protein expression. Future studies determining autophagic flux in EAT in the setting of obesity are needed.

#### Autonomic Dysfunction and EAT

The autonomic nervous system (ANS) has ganglionated plexi (GPs) within the heart located in the epicardial fat pads that can modulate cardiac autonomic nerve input (Zhou et al., 2014). Vagal stimulation is modulated through multiple cardiac GPs before reaching the sinus and atrio-ventricular (AV) nodes (Zhou et al., 2014). Autonomic dysfunction leading to atrial fibrillation has already been well-described. The parasympathetic system seems to contribute significantly to AF in young patients with otherwise normal hearts (Coumel, 1996). Firstly, there is significant vagal innervation of the atrial muscle sleeves extending into the pulmonary veins (Zipes and Knope, 1972). Vagal tone is mediated via acetylcholine which binds to the muscarinic receptor. This in turn can directly activate the G-protein-gated atrial K+ channel (I_KACh_) leading to hyperpolarization and indirectly inhibition of cyclic adenosine monophosphate (cAMP; Harvey and Belevych, 2003). This results in shortening of the atrial action potential duration with increased spatial heterogeneity (Rosenshtraukh et al., 1989) allowing for atrial fibrillation. Furthermore, GPs can be located adjacent to areas of targeted for pulmonary vein isolation (PVI). PVI can result in vagal denervation of the left atrium (Yang et al., 2011). Complex fractional atrial electrograms are commonly located at sites of GPs and ablation of complex fractionated atrial electrograms has been associated with AF ablation success in some studies although this has been controversial (Nademanee et al., 2004). Conversely, the sympathetic system also can incite atrial fibrillation as well as ventricular arrhythmias by directly triggering β-adrenergic mediated increasing of intracellular Ca^2+^ (Francis, 1988). Given GPs are located in the epicardial fat pads, EAT may play a strong role in regulating autonomic tone. It is possible that in the setting of obesity, EAT autonomic signals become dysregulated and allow for arrhythmia. Disproportional increase of pericardial fat may be associated with abnormal augmentation of autonomic tone, which might lead to increased VT/VF and mortality (Wu et al., 2015).

### Intramyocardial Fat

There have been numerous links made between intramyocardial fat and arrhythmias. Fibrofatty replacement of myocytes is one of the hallmarks of arrhythmogenic right ventricular cardiomyopathy (ARVC) and is thought to constitute the nidus for re-entry ventricular arrhythmias (Bauce et al., 2005). The characteristic adiposis associated with ARVC seems to be part of a non-specific repair process similar to the one that takes place after ischemia (Samanta et al., 2016). It is thought that after myocardial infarction (MI), patients are at risk of developing ventricular arrhythmias due to scar tissue formation. Yet, not all patients develop VT after MI despite the presence of intramyocardial collagen (Pouliopoulos et al., 2013). This may be explained in part by the greater electrical resistance of adipose tissue compared to myocardium or collagen, which may promote reentry (Rudy et al., 1979; Fallert et al., 1993; Pouliopoulos et al., 2013). This is also supported by animal studies showing that intramyocardial adipose tissue can impede myocardial conduction and attenuate both electrogram amplitude and slope to a greater extent than collagen (Pouliopoulos et al., 2013). However, conflicting findings have been shown in an *in-silico* computational model, where adipose tissue was found to be less arrhythmogenic than fibrotic tissue in terms of percentage of non-conducting tissue needed to induce the arrhythmias (De Coster et al., 2018). Other mechanisms for arrhythmogenesis should also be considered, such as the presence of gap junctions in adipose tissue, that as mentioned before may lead to aberrant signal propagation (Linck et al., 1974).

Intramyocardial fat has also been associated with supraventricular arrhythmias (Haemers et al., 2017). These fibro-fatty infiltrations in human atrial biopsy samples have also been associated with increased inflammation given findings of small lymphoid aggregates composed mainly of CD8+ cytotoxic effector T cells (Haemers et al., 2017) which have been reported to have a pathogenic role in adipose tissue inflammation in the setting of obesity (Nishimura et al., 2009; Pouliopoulos et al., 2013; Haemers et al., 2017).

The relationship between EAT and the myocardium is likely to be bidirectional. In an animal model of AF using rapid atrial pacing, there was an increase in expression of genes that favor adipocyte differentiation and infiltration to the right atrium, leading to increases in adipose tissue mass (including EAT) and lipid accumulation (Chilukoti et al., 2015). A recent study also found that progenitor cells located in the epicardium can undergo epithelial-to-mesenchymal transition process and give origin to adult EAT adipocytes, and this process is driven by atrial natriuretic peptide (ANP) secreted by the atrial myocardium (Suffee et al., 2017). Recently, NLRP3 inflammasome activation in atrial cardiomyocytes was shown to promote AF, likely due to increased calcium release from the ER and increased production of pro-inflammatory cytokines (Yao et al., 2018). This is relevant given that in the setting of obesity the NLRP3 inflammasome is activated (Haneklaus and O'Neill, 2015). So it is possible that inflammasome activation can contribute to arrhythmia pathogenesis.

### Remote Adipose Tissue

Remote fat cells have numerous endocrine effects. The interplay between the endocrine function of fat and the heart has not been fully elucidated. However, there are a couple of examples highlighting how increased fat burden can lead to arrhythmia. Obesity is characterized by a change in cardiac metabolism (Lopaschuk et al., 2007). Under normal conditions free fatty acids are the major source of energy for the heart given its high demand. In the setting of obesity, there is increased circulating free fatty acids, which may lead to fatty acid infiltration of myocardial tissue, increases in fatty acid oxidation, insulin resistance, and glucose intolerance, all of which decreases cardiac metabolic efficiency (Lopaschuk et al., 2007). Insulin resistance provokes systemic hyperglycemia which can result in myocardial injury (glucotoxicity; Ebong et al., 2014). Classically, obesity is also associated with low grade chronic inflammation. Visceral fat has higher levels of pro-inflammatory cytokines compared to EAT in CAD patients, leading some to think that part of the inflammatory effects may be systemic, arising from sources such as visceral fat and not just epicardial or local fat (Cheng et al., 2008).

Adipocytes secrete leptin, a peptide hormone which regulates sympathetic outflow. In a canine MI model of left anterior descending artery occlusion, leptin injection into the left stellate ganglion was associated with increased ventricular arrhythmia incidence and decreased action potential duration and duration dispersion compared to controls (Yu et al., 2018). Adipocyte fatty acid-binding protein (FABP4), which is predominantly expressed in adipose tissues, is known to depress shortening amplitude as well as intracellular systolic peak Ca^2+^ in a dose-dependent manner in isolated rat cardiomyocytes. Both the calcium dysregulation effect as well as the cardio-inhibitory effect leading to heart failure can lead to atrial/ventricular arrhythmias (Lamounier-Zepter et al., 2009). Also, increased leptin levels can lead to increased aldosterone secretion, systemic inflammation, endothelial dysfunction, increased vascular stiffness, hypertension, and cardiac hypertrophy, all of which could contribute to the pathogenesis of AF (Huby et al., 2015). Obesity-related adrenergic activation in particular may allow for more triggered arrhythmia. Overexpression of angiotensin I receptor and angiotensin converting enzyme related carboxypeptidase (ACE2) can lead to AV block and spontaneous ventricular tachycardia (Verheule et al., 2004).

**Figure 2:**
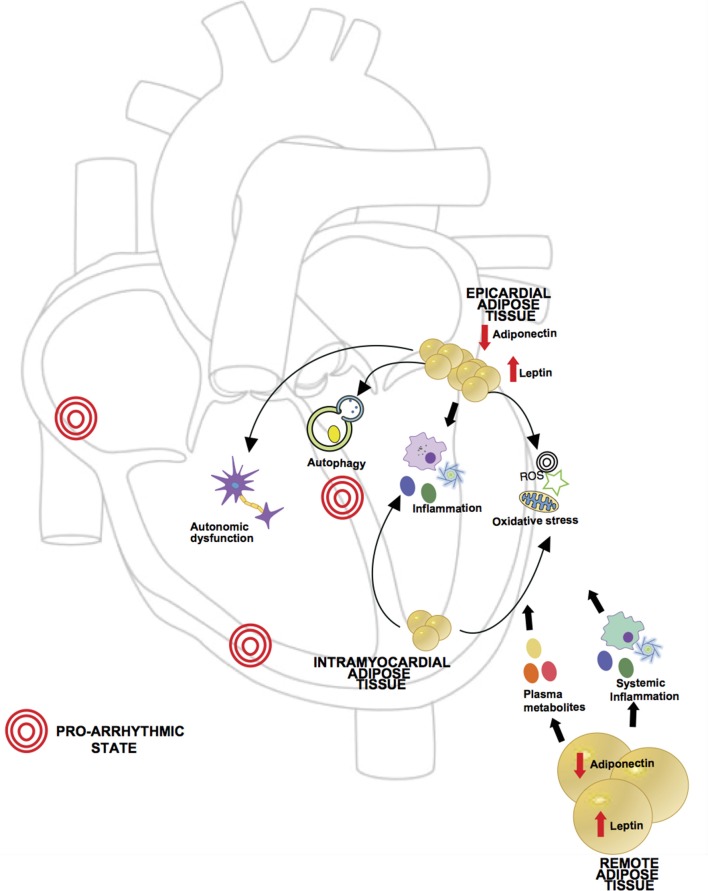
Proposed mechanisms of obesity in pathogenesis of arrhythmia. Obesity is characterized by a dysregulated state of adipose tissue, leading to an increase in pro-inflammatory adipokines (i.e., leptin) and decrease in anti-inflammatory adipokines (i.e., adiponectin), creating a chronic inflammatory state which has been related to arrhythmia development. Levels of circulatory metabolites are also increased such as free fatty acids and glucose leading to cardiac glucotoxicity and increased fatty acid oxidation. Given the anatomic location, local adipose tissue can exert direct effects in myocardial function. In obesity, epicardial adipose tissue (EAT) has been shown to have increased macrophage infiltration and higher levels of pro-inflammatory and pro-fibrotic cytokines. Excess lipids and hyperglycemia can induce mitochondrial dysfunction in adipocytes causing excessive production of reactive oxygen species (ROS). In setting of obesity aberrant autophagy activation in adipocytes can lead to degradation of adiponectin, contributing to the pro-inflammatory state. Also, ganglionated plexi of the autonomic nervous system are located in epicardial fat pads and in setting of obesity there may be an abnormal augmentation of autonomic tone. Intramyocardial fat affecting atrial tissue has also been described in association with supraventricular arrhythmias, likely through oxidative stress and perpetuation of inflammation.

In summary, remote adipose tissue can cause arrhythmia via a multitude of mechanisms from total body adipose tissue load related hemodynamic changes, neurohormonal mediated cardiac structural remodeling, adrenergic system activation and chronic low grade inflammation. Figure 2 reviews the different possible mechanisms implicated in arrhythmogenesis previously described.

### Dietary Fat

Independent of obesity-induced cardiac structural changes mentioned earlier (i.e., biventricular enlargement/hypertrophy, diastolic/systolic dysfunction), increased dietary fat burden alone can lead to arrhythmia. For example, even a short period of HFD in gerbils can lead to radical inflammation and apoptosis causing cardiac remodeling (Sahraoui et al., 2016). Rats fed a high fat diet had an increased incidence of malignant ventricular tachyarrhythmias (Liptak et al., 2017) in the setting of induced ischemia. Furthermore, atria from rats fed high fat diet showed upregulation of transforming growth factor-β1 and matrix metalloproteinase-2 (atrial fibrosis pathway proteins), gap junction remodeling with alterations and distribution of connexin 40 (Cx40) and Cx43, broadened interstitial space, and myocyte disarray, and increased apoptotic cell death (Meng et al., 2017). In guinea pigs, HFD produces alteration of atrial cell electrophysiology favilitating the development of reentrant arrhythmias (Aromolaran et al., 2016).

In humans, hypercholesterolemia has been associated with QT prolongation, offering yet another mechanism for potential arrhythmia (el-Gamal et al., 1995). When studying the effect of palmitic, stearic, and oleic free fatty acids on sheep atrial myocytes, it was found that stearic acid disrupts t-tubular and induces inhibition of I_Ca−L_ and I_TO_ ionic currents in sheep atrial myocytes. This could result in abbreviation (I_Ca−L_) or a prolongation (I_TO_) of atrial myocyte action potential duration and thus play role in arrhythmogenesis (O'Connell et al., 2015). This also highlights that not all dietary lipid is the same. In pig ventricular myocytes, for example, dietary fish oil prevents calcium overload and reduces incidence of norepinephrine induced triggered activity (Berecki et al., 2007). Clinical trials in humans looking at the utility of fish oil in the prevention of arrhythmia in various cohorts, however have been contradictory. In the Alpha-Omega trial, low-dose EPA-DHA, or ALA did not reduce the rate of major cardiovascular events, but secondary analysis of the data showed a reduction in arrhythmias in the fish oil groups (Kromhout et al., 2010). In the SOFA trial, omega-3 poly unsaturated fatty acids from fish oil did not significantly protect against ventricular arrhythmia in patients with implantable cardioverter defibrillators (Brouwer et al., 2006).

High fat diet (HFD) has also been studied in AF. In a study using a sheep model of HFD-induced obesity, there was infiltration of the posterior left atrium by EAT associated with reduced endocardial voltage in this region compared to lean sheep (Mahajan et al., 2015). HFD was associated with biatrial endocardial remodeling, conduction abnormalities, increased expression of TGF- β_1_ in the left atrium and interstitial atrial fibrosis leading to increase frequency and duration of AF (Mahajan et al., 2015). In mice, HFD-induced obesity has been shown to be related to increased LA fibrosis and higher vulnerability of developing AF (AF was able to be induced in 50% of animals that were treated with HFD vs. 0% in the control group; Kondo et al., 2018). Further, studies are needed to observe the impact of diet modifications and weight loss in animals in reversing alterations in electrophysiology.

## Therapeutics

In the case of obesity, a single mechanism likely does not account for the link between adipose tissue and arrhythmia. Rather, it is the activation of multiple pathways and effects of adipose tissue that make the myocardium more prone to arrhythmia. Treating this requires a multidisciplinary approach with upstream incorporation of risk factor modification (RFM) and lifestyle modifications. In a study of 1425 consecutive patients offered weight management, those with >10% weight loss had 6 fold increased likelihood of arrhythmia free survival over 5 years compared to those with less weight loss (Pathak et al., 2017). Similar data has been reported in an AF ablation cohort. In 281 consecutive patients undergoing AF ablation, patients who opted for RFM had greater reductions in weight, blood pressure and lipid profiles. In both single procedure and multiple procedure drug unassisted cases, patients opting for RFM had significant arrhythmia free survival compared to controls. On multivariate analysis, RFM was an independent predictor of arrhythmia free survival (Pathak et al., 2014). An even more provocative finding is that when RFM is implemented rigorously, achieved via face to face consultation for behavioral change as it relates to weight management and physical activity), RFM turned out to be a very cost effective measure with an increase of 0.193 quality-adjusted life years in those patients with symptomatic AF who opted for RFM compared to those who opted out. This is a cost saving of $12,094 (Pathak et al., 2017). How this relates to ventricular arrhythmia, remains to be seen but RFM should be a cornerstone in the treatment of arrhythmia, especially atrial fibrillation.

## Future Directions

The relationship between obesity and the development of arrhythmias is exciting, but further work to clarify the mechanisms and possible therapeutic interventions is needed. Identification of the proarrhythmogenic mechanisms elicited by obesity will involve basic, translational and clinical research. Clinical arrhythmias may be multifactorial in nature. Thus, teasing apart the mechanisms that drive an arrhythmia in any individual patient will likely require further work.

One of the most interesting areas is the process of the whitening of EAT. It is possible that in setting of lipid overload, ROS production becomes uncontrollable leading to inflammation and whitening of EAT (Dozio et al., 2014). However, given EAT's thermogenic function where ROS production is high at baseline, there may be a transition point at which ROS production may become excessive having pathogenic effects on the heart. Targeting EAT activity and ROS production could be leveraged to treat or prevent arrhythmias.

As discussed previously, EAT affects the adjacent myocardium by both paracrine mechanisms as well as by direct infiltration. However, mechanisms of how EAT infiltrates the myocardium remains poorly understood. It seems like one of the most common characteristics found in the studies mentioned above are the inflammatory properties of the secretome from EAT in the setting of obesity that leads to myocardial fibrosis. Atrial fibrosis is a feature of AF, and activin A, a member of the TFG-β superfamily has been associated with the development of atrial fibrosis (Venteclef et al., 2015). Importantly, activin A has been shown to be upregulated by angiotensin-II in cardiomyocytes and atrial fibroblasts in non-obese mice (Wang et al., 2017). EAT from CAD patients has been shown to have higher expression of angiotensinogen than control patients. Thus, it is possible that EAT activation of the renin-angiotensin system may be a key player in the stimulation of atrial fibrosis as a substrate for AF.

Another key player to target would be TGF- β _1_. As mentioned earlier, sheep models fed a HFD have higher TGF- β _1_ levels in their atrium. *In vitro*, human atrial myofibroblast tissue treated with TGF- β _1_ showed increased collagen Ia2 and fibronectin synthesis (both markers of active fibrosis) as well as increased autophagy activation possibly leading to atrial fibrosis._._Thus, exploring how fat affects TGF- β _1_ and potential mechanisms to inhibit this pathway may offer further treatment options.

Increased ROS production and inflammation can lead to ER stress. SERCA proteins are calcium pumps that transport Ca^2+^ from the cytoplasm into the ER (Kang et al., 2016). SERCA dysfunction leading to decreased or increased cytoplasmic calcium levels, has been related to ER stress and arrhythmia (Erkasap, 2007; Kang et al., 2016). Determining the factors involved in the development of ER stress and the regulation of expression of SERCA proteins in myocardium and adipose tissue in the setting of obesity may be key. These findings underline the need to better understand the pathogenic effects of obesity in arrhythmogenesis and the role of inflammation, oxidative stress, ER stress and autophagy, as well as the potential therapeutic interventions that can prevent electrophysiological alterations.

### Research Models

Small animal models (such as mice or rats) are valuable for the study of cardiac physiology given the lower maintenance cost, easier handling, cost effectiveness and possibility for genetic manipulation (Milani-Nejad and Janssen, 2014). Techniques for assessing cardiac function and electrophysiology measures are well-established for rodents. Despite being the most common *in vivo* model used, differences between murine models and human patients can make the generalizability of the data obtained from murine experiments on action potentials, cardiac contraction, cardiomyocytes characteristics and protein expression difficult (Nerbonne, 2004; Milani-Nejad and Janssen, 2014). Specifically, for the study of arrhythmias, baseline heart rates for rodents are greater than humans, which leads to a rapid repolarization and lack of plateau phase in the action potentials of ventricular myocytes observed in large animals (Nerbonne, 2004). Guinea-pigs have a more similar action potential wave given similarities in ionic currents contributing to repolarization (Bosch et al., 1998). Rabbits have also been used for the study of cardiac diseases given that rabbit myocardium shares more similarities with the human myocardium than small rodents (Milani-Nejad and Janssen, 2014); but the action potential is still faster than humans (Szél et al., 2011). Larger animal models share more similarities with human physiology, but their maintenance and costs can be challenging. Moreover, the contractile and relaxation kinetics in larger animal models remain slightly faster than humans (Milani-Nejad and Janssen, 2014), and the ion channel distribution in the different species is unknown (Schram et al., 2002; Milani-Nejad and Janssen, 2014).

Specific rat models which available for study would include those with mutations in the calcium channel SCN5A in mice which is associated with Brugada syndrome (Milan and MacRae, 2005) and mice with human Na_V_1.5 variant with a mutation in the anesthetic-binding site that produces an incomplete Na^+^ channel inactivation (F1759A-Na_V_1.5), who develop spontaneous AF induction (Wan et al., 2016). How these arrhythmia prone models respond to obesity may offer further insight into the link between arrhythmia and obesity.

Possible human clinical areas of research would be rigorous quantification of different types of adipose tissue. This would allow risk stratification for hard clinical outcomes such as predicting new AF development as well as recurrence after ablation, prediction of ventricular arrhythmias after MI, risk stratification for need for implantable cardioverter defibrillator in various disease where risk is not clear (e.g., asymptomatic, gene positive, phenotype negative hypertrophic cardiomyopathy patients).

Recently human induced pluripotent stem cell derived cardiomyocytes from disease patients have been shown to be a promising method to study cardiac arrhythmias, especially in arrhythmias related to genetic mutations (Garg et al., 2018). Pluripotent stem cell derived cardiomyocytes can be used to study channelopathies by modeling genetic syndromes in an *in vitro* dish (Garg et al., 2018). Even though the relevance of pluripotent stem cells in arrhythmias with multifactorial etiologies (including metabolic syndrome) is limited, endothelial cells derived from HFD mice exhibit signs of endothelial dysfunction compared to regular diet mice (Gu et al., 2015). Further studies are needed to delucidate if pluripotent stem cell derived cardiomyocytes from obese animals or patients can be used to model arrhythmias.

## Conclusion

There has been an epidemic level rise in obesity. Emerging evidence suggests that obesity promotes atrial and ventricular arrhythmias. However, the function of adipose tissues is multifaceted and its interplay with the heart is even more complex. Future studies examining the connection between adipose tissues and arrhythmias are urgently needed. Numerous adipose tissue depots, such as intramyocardial, local and/or remote may play integral roles in the regulation of inflammation, oxidative stress, autophagy, autonomic tone, mitophagy, propagation of aberrant signals, creation of potential re-entry circuits and cardiomyocyte death. Upon elucidation of the mechanisms by which arrhythmias are incited, it may be possible to identify high value therapeutic targets.

## Author Contributions

MP and KM are co-first authors. MP, KM, JC, and JL contributed and reviewed and approved the final manuscript. All co-authors contributed and reviewed and approved the final manuscript.

### Conflict of Interest Statement

The authors declare that the research was conducted in the absence of any commercial or financial relationships that could be construed as a potential conflict of interest.
